# Knowledge, attitudes and acceptance of voluntary medical male circumcision among males attending high school in Shiselweni region, Eswatini: a cross sectional study

**DOI:** 10.1186/s12889-023-15228-3

**Published:** 2023-02-16

**Authors:** Mirriam Hlelisani Shezi, Boikhutso Tlou, Saloshni Naidoo

**Affiliations:** 1 Senior Radiographer, Nhlangano Health Centre, Shiselweni, Eswatini Eswatini; 2 Masters in Public Health Graduate, Discipline of Public Health Medicine, School of Nursing and Public Health, University of KwaZulu-Natal, Durban, South Africa; 3 Lecturer in Biostatistics, Discipline of Public Health Medicine, School of Nursing and Public Health, University of KwaZulu-Natal, Durban, South Africa; 4 Discipline of Public Health Medicine, School of Nursing and Public Health, University of KwaZulu-Natal, Durban, South Africa

**Keywords:** Voluntary medical male circumcision, Acceptance, Knowledge, Attitudes, Adolescents, Eswatini

## Abstract

**Background:**

In countries such as Eswatini, where there is a high HIV prevalence and low male circumcision the World Health Organization and the Joint United Nations Programme for HIV/AIDS recommend infant and adult circumcision be implemented. The aim of this study was to assess the knowledge, attitudes and acceptability of voluntary medical male circumcision amongst males attending high school in Eswatini.

**Methods:**

An observational cross-sectional study was conducted during February and March of 2018 amongst 407 young males (15–21 years) attending Form 4, in nine high schools in the Shiselweni region of Eswatini using a self-administered questionnaire of 42 close ended questions. Sociodemographic details, circumcision status, acceptance of voluntary medical male circumcision, knowledge and attitude scores analysed in Stata® 14 statistical software were described using frequencies, medians and ranges respectively. Bivariate and multivariate linear regression was used to assess the impact of independent variables on circumcision status and acceptance of voluntary medical male circumcision. The level of statistical significance was p < 0.05.

**Results:**

Amongst the 407 high school-going males, 48.98% (n = 201) reported being circumcised. The majority of the adolescents (75.74%; n = 306) were knowledgeable about voluntary medical male circumcision. However, an even larger majority (84.90% (n = 343) had a negative attitude towards it. In the multivariate logistic regression analysis, having parented their own children (aOR: 3.55; 95%CI: 1.2–10.48), and having circumcised friends (aOR: 3.99; 95%CI: 1.81–8.84) were significantly associated with being circumcised. Neither knowledge nor attitude were associated with the acceptability of voluntary medical male circumcision.

**Conclusion:**

In Eswatini male high school students are knowledgeable about voluntary medical male circumcision but have a negative attitude towards it. Having parented their own children, and having circumcised friends influenced being circumcised.

## Background

Human Immunodeficiency Virus (HIV) and Acquired Immune Deficiency Syndrome (AIDS) are a global public health problem. There were an estimated 37.7 million people living with HIV at the end of 2020, over two thirds of whom (25.4 million) are in Africa [1]. Despite the significant impact, new infections in this region have declined by 38% since 2010 [2]. However, in order to reach the new proposed global 95–95–95 targets set by Joint United Nation Programme on HIV/AIDS (UNAIDS), there is still a need to redouble the efforts to avoid the worst-case scenario of a half million excess HIV-related deaths in Sub-Saharan Africa (SSA), increasing HIV infections due to HIV service disruptions during COVID-19, and the slowing public health response to HIV [1]. Eswatini, in the SSA region has the highest HIV prevalence at 27% [3].

In 2007, the World Health Organisation (WHO) and UNAIDS recommended voluntary medical male circumcision (VMMC) always be part of the comprehensive HIV prevention package[4]. This was after the Randomised Clinical Trials (RCTs) done in Africa showed a 60% HIV incidence reduction in circumcised men compared to the uncircumcised [5–7]. Eswatini is one of the 14 countries advised to include VMMC in their HIV prevention package[8]. The Eswatini Ministry of Health’s Male Circumcision Strategic and Operational Plan 2014–2018 adopted an implementation strategy that called for VMMC to be scaled up to 80% among males aged 10–29 years and 55% among males aged 30–34 years [9]. ,[10, 11].

Several studies have found VMMC is accepted by adults in SSA [12–14]. Since the UNAIDS and WHO recommended VMMC to reduce the HIV epidemic, Eswatini has put in more effort to meet the recommendation by even introducing Soka Uncobe campaign[14]. Unfortunately, it was less effective due to a concoction of long-entrenched local traditions, false rumours, economic pressures and gender imbalances [15]. Swatis currently do not circumcise in the cultural context, although it was once a coming-of-age ritual until the mid – 1800s. That is another factor that was misjudged how long it takes to institute a procedure in such non-circumcising culture [15]. A recent study done in Eswatini to assess factors associated with the parent’s decision on Early Infant Male Circumcision (EIMC) showed mothers had good knowledge of male medical circumcision (MMC), and accepted it [16]. Nevertheless, adolescents have different social concerns and risks, and their knowledge and attitudes are unknown [17, 18]. Voluntary medical male circumcision in adolescents and youth can reduce the risk of HIV infection and is cost-effective, as the prevalence is low in their age group [19]. Therefore, there is a need to assess their knowledge and attitudes and to understand the impact these elements have on the acceptability of VMMC amongst young males [20].

## Methods

### Study design

This study was an observational cross-sectional study with descriptive and analytical components.

### Study setting

The study was conducted in high schools in the Shiselweni region of Eswatiniin southern Africa [3]It is divided into four administrative regions. The estimated population of Eswatini in 2017 was 1 119 375, 49% of whom are under 20 years of age [21]. Shiselweni is the most impoverished of the country’s four regions with an HIV prevalence of 25.9% [3]. Approximately 6.1% of males had completed secondary education, and 2.5% in the region had reached tertiary education by 2015 [22].

### Population and sampling

The study population comprised all male students in Form 4, attending nine selected high schools out of the 54 high schools in Shiselweni region, Eswatini. The study was conducted during February up to March 2018.

A complete sampling frame was used where all of the 54 high schools in the Shiselweni region were listed and grouped as urban versus rural. All of the four urban high schools were automatically included in the study, and 10% (n = 5) of the rural high schools were randomly selectedThus it was estimated that there were approximately 2320 males in the 54 schools in Shiselweni. Assuming a male circumcision prevalence of 40% with a precision of 5%, the minimal sample size that was required was 318 participants [23]. A total number of 436 participants were recruited in this study period and 29 of the questionnaires were spoiled. Only 407 were analysed.

Male students were invited to participate in the study, and the study was explained to them. Information and consent forms explaining the details of the study were distributed to the adolescents for their parents or guardians to read and sign if they understood and agreed to their sons’ participation in the study. Parents had to give consent, and participants below 18 years old had to assent.

### Measures

#### Demographics

Demographic information on Table 1 was collected .

**Table 1:** Demographic profile and sexual behaviour of young males in form four in Shiselweni region, Eswatini in 2018 (n = 407)

Variable	Frequency (%)
**Age (mean; 95%CI)**	18.46 (18.18–18.74)
**Birth Region**	
Hhohho	29 (6.58)
Shiselweni	310 (76.59)
Manzini	47 (12.35)
Lubombo	17 (3.79)
Other	4 (0.69)
**Population Group**	
African	399 (98.75)
Coloured	8 (1.25)
Indian	0
White	0
**Religion**	
Christian	369 (91.15)
Muslim	6 (1.44)
Hindu	2 (0.87)
Traditional	30 (6.53)
Other	0 (0)
**Place of residence**	
Boarding school	36 (10.25)
At Home	323 (79.65)
Other (foster homes, rented flat, live with relatives)	48 (10.1)
**With whom do you live?**	
Both Parents	126 (42.13)
Mother Only	98 (29.58)
Father only	25(7.04)
Grandmother/ grandfather	65 (18.97)
Other (uncle, aunt, siblings)	9 (2.28)
**Number of people in your household (n;%)**	
1	4 (0.9)
2	18 (3.92)
3	31 (8.17)
4	85 (22.29)
5 or more	267 (64.72)
**Household income (n; %)**	
E500- E1000	55 (13.44)
E1000- E 5000	47 (9.75)
E 5000- E 10,000	28 (7.11)
E 10,000- and above	23 (5.56)
Do not Know	254(64.14)
**School location (n; %)**	
Rural	286 (66.27)
Urban	121 (33.73)
**Sexually active**	
Yes	239 (57.28)
No	168 (42.72)
**If Yes, at what age did you become sexually active**	
10–15 years	49 (17.62)
15–20 years	186 (80.95)
21 and above	4 (1.43)
**Do you use a condom**	
Always	126 (51.51)
Sometimes	85 (36.34)
Never	28 (12.15)
**Have you parented any children**	
Yes	29 (6.89)
No	378 (93.11)
**How many children do you have**	
1	20 (65.17)
2	8 (30.82)
3 or more	1 (4.01)

Note: 1E = Lilangeni currency (Swaziland) = **1ZAR** = 0.0528941 **GBP**

Note ^1^ the weighted percentages of the frequencies

#### Knowledge

Participants were asked to report their level of understanding information about male circumcision by indicating the extent to which they agreed with the statements on a pre-determined Likert scale (Strongly disagree, disagree, neutral, agree, strongly agree) as shown in Table 2.

**Table 2:** Frequency distribution table of knowledge and attitudes toward voluntary medical male circumcision in young males in Shiselweni high schools, Eswatini, 2018 (n = 407)

Knowledge Variable	Frequency (%)	
Circumcision only reduces female to male HIV transmission		
Agree	102 (25.06)	
Neutral	64 (15.72)	
Disagree	241 (59.21)	
**Circumcised men are 100% protected from HIV**		
Agree	20 (4.91)	
Neutral	30 (7.37)	
Disagree	357 (87.71)	
**Circumcised men do not need to use a condom**		
Agree	11 (2.70)	
Neutral	16 (3.93)	
Disagree	380 (93.37)	
**Only sexually active men need to circumcise**		
Agree	72 (17.69)	
Neutral	42 (10.32)	
Disagree	293 (71.99)	
**Circumcision can reduce the risk of cancer in men**		
Agree	284 (69.95)	
Neutral	54 (13.30)	
Disagree	68 (16.75)	
**Circumcision reduces the risk of sexually transmitted Infections**		
Agree	319 (78.38)	
Neutral	39 (9.58)	
Disagree	49 (12.04)	
**Circumcision improves penile hygiene**		
Agree	294(72.24)	
Neutral	62 (15.23)	
Disagree	51 (12.53)	
**Circumcision at adolescence may result in fertility problems**		
Agree	61 (15.06)	
Neutral	77(19.01)	
Disagree	267 (65.93)	
**VMMC decreases sexual satisfaction**		
Agree	104 (25.55)	
Neutral	99 (24.32)	
Disagree	204 (50.12)	
**Circumcised men have more sexual feelings than uncircumcised men**		
Agree	60 (14.74)	
Neutral	121 (29.73)	
Disagree	226 (55.53)	
**Circumcised men enjoy sex more than uncircumcised men**		
Agree	165 (40.54)	
Neutral	116 (28.50)	
Disagree	126 (30.96)	
**Women prefer circumcised men than uncircumcised**		
Agree	199 (48.89)	
Neutral	123 (30.22)	
Disagree	85 (20.88)	
**Circumcision is un-Godly**		
Agree	95 (23.34)	
Neutral	115 (28.26)	
Disagree	197 (48.40)	
**Circumcision is un-Swati**		
Agree	76 (18.67)	
Neutral	125 (30.71)	
Disagree	206 (50.61)	
**Circumcision proves manhood**		
Agree	143 (35.14)	
Neutral	120 (29.48)	
Disagree	144 (3 5.38)	
**Circumcision makes one incomplete**		
Agree	127 (31.20)	
Neutral	76 (18.67)	
Disagree	204 (50.12)	
**The tip of the penis must always be covered**		
Agree	94 (23.10)	
Neutral	110 (27.03)	
Disagree	203 (49.88)	
**VMMC is old fashioned and must not be done in modern society**		
Agree	67 (16.46)	
Neutral	59 (14.50)	
Disagree	281 (69.04)	
**The foreskin is used as raw material for certain spice**		
Agree	71 (17.57)	
Neutral	71 (17.57)	
Disagree	262 (64.85)	

Note ^1^ the weighted percentages of the frequencies

#### Attitudes

The attitude questions were to indicate the extent to which the participants agreed with the statements on a pre-determined Likert scale as in knowledge. The attitude questions covered the beliefs, feelings and perceptions.

The knowledge and attitude questions were developed using the following Health Belief Model’s constructs: Perceived susceptibility, Perceived severity (threat of HIV), Perceived benefits, Perceived barriers and Cues to action.

#### Acceptance

The participants were asked to report their circumcision status and their future willingness to get circumcised.

#### Data analysis

Stata®14 statistical software (StataCorp.2014. Release 14 College Station, TX: StataCorp LP) was used to analyse the data. Categorical and continuous data were summarized using frequency distributions and percentages and median, means and 95% confidence intervals, respectively. The knowledge questions were scored according to the Likert scale which in the following categories: strongly agreed, agreed, neutral, disagreed, strongly disagreed. The categories were then collapsed to agreed, neutral, and disagreed. Knowledge scores were generated by summing together the correct responses to the knowledge questions. The attitude questions were scored by allocating a point each to the positive attitudes. The knowledge and attitudes scores were categorised around the median. A score equal to or above the median was defined as having knowledge and a positive attitude respectively. There were two dependent variables under study: [1] presence of circumcision and [2] acceptability of VMMC (intention to be circumcised). To test for associations of knowledge and attitudes with the presence of circumcision, adolescents who were circumcised were the focus of the analysis. To test for associations of knowledge and attitudes with acceptability of VMMC, adolescents who intended to circumcise were the focus of the analysis. Bivariate analysis between the study outcomes and all independent variables was conducted. All predictors that were found to be statistically significant (p < 0.05) on bivariate analysis were then put into the multivariate models using multilevel logistic regression to identify the factors influencing the presence of circumcision and the acceptability of VMMC (intention to be circumcised). Multilevel regression analysis was used to account for the clustering of students within clusters of higher-level units, being the schools.

## Results

### Demographic profile of the study participants

Four hundred and seven high school males participated in the study in the age range from 15 to 21 years, with a median age of 18.46 years. A greater proportion (n = 310; 76.59%) of the participants were born in the Shiselweni region. The majority (n = 323; 79.65%) of the participants lived at home in 2018 and 42.13% (n = 126) of them lived with both parents (Table 1).

### Knowledge of voluntary medical male circumcision among male adolescents

Most participants disagreed, with statements that circumcision only reduces female to male HIV transmission (n = 241; 59.21%), circumcised men are 100% protected from HIV (n = 357;87.71), circumcised men do not need to use a condom (n = 380; 93.37%), only sexually active men need to circumcise (n = 293, 71.99%) and circumcision in adolescence may result in fertility problems (n = 267; 65.93%).

Most participants agreed with statements that circumcision can reduce the risk of cancer (n = 284; 69.95), circumcision prevents the risk of sexually transmitted disease (n = 319; 78.38%) and circumcision improves penile hygiene (n = 294; 72.24%). **(**Table 2**).** Participants were scored for their knowledge responses and the median knowledge score was 31 (Range: 18 to 42). Those participants who scored equal to and above this score were considered knowledgeable (n = 306; 75.74%).

### Attitudes of high school attending males towards voluntary medical male circumcision

Half of the participants (n = 204; 50.12%) disagreed that male circumcision decreases sexual satisfaction, and a slight majority (n = 226; 55.53%) disagreed that circumcised men have more sexual feelings than uncircumcised men. A large minority (n = 165; 40.54%) agreed that circumcised men enjoy sex more than uncircumcised men (Table 2). Near half of the participants (n = 199; 48.89%) agreed that women preferred circumcised men over uncircumcised men. Most participants disagreed that circumcision is un-Godly (n = 19748.4%) and un-Swati (n = 206; 50.61%) (Table 2). Among the participants, about half of the participants (n = 203; 49.88%) disagreed that the tip of the penis must always be covered. We further investigated if they thought male circumcision in adolescents was very painful and may lead to death, and 61.82% (n = 251) disagreed. Most participants (n = 281;69.04%) disagreed that male circumcision is old fashioned and must not be done in modern society. The majority (n = 262;64.85%) of the participants did not believe the myth that the foreskin is used as a raw material for certain spice (Table 2). The median attitude score was 40.0 (Range:20–60). Those participants who scored equal to and above the median score (n = 61; 15.1%) were considered to have had a positive attitude toward VMMC.

### Sexual behaviour amongst the young males

The majority of participants (n = 238; 57.39%) were sexually active. Furthermore, an even larger proportion (n = 192; 80.95%) of them of them had started between the age of 15–20 years. A little over half of those (n = 144; 51.51%) who were sexually active reported that they always used a condom when engaging in sexual intercourse. A few of the participants (n = 29; 6.89% ) were fathers (Table 1).

### Acceptability of voluntary medical male circumcision among male participants

Amongst the participants, a slight majority (n = 206; 51.02%) were uncircumcised, and 23.43% of those uncircumcised were willing to be circumcised when they got a chance. However, 31.81% (n = 17) of them were not yet sure when they were going to get circumcised. Table 3 shows that the majority (68.5%) of the participants had brothers (n = 158; 59.11%) and friends (n = 280; 71.96%) who were circumcised.

**Table 3:** Circumcision status and acceptability of voluntary medical male circumcision among participants attending form 4 in Shiselweni high schools, Eswatini, 2018 (n = 407)

Variable	Frequency (%)^1^
Are you circumcised?	
Yes	201 (48.98)
No	206 (51.02)
**Are any of your family members circumcised?**	
Yes	276 (68.50)
No	92 (21.99)
Do not know	39 (9.51)
**Who is circumcised in the family?**	
Grandfather	3 (0.70)
Father	40 (14.60)
Brother	163 (59.11)
Cousin	46 (16.34)
Other ( uncle, nephew)	25 (9.25)
**Do you have friends who are circumcised?**	
Yes	287 (71.96)
No	60 (14.36)
Not sure	60 (13.68)
**If Not circumcised, given a chance would you be circumcised?**	
Yes	52 (23.43)
No	87 (38.39)
Not Sure	67 (38.18)
**If yes, when?**	
Next school vacation	17 (30.25)
When I finish school	9 (1.94)
About to get married	9 (18.54)
Not sure	17 (31.81)

Note ^1^ the weighted percentages of the frequencies

### Factors associated with the presence of circumcision and acceptability of voluntary medical male circumcision

Adolescents who lived with both parents were less likely to have been circumcised (cOR: 0.43; 95%CI: 0.23–0.79) on bivariate analysis, and this association persisted on multivariate analysis (aOR: 0.35; 95%CI: 0.18–0.69). Young males who had parented a child (aOR: 3.55; 95%CI: 1.22–10.48) and those who had friends who had been circumcised (aOR: 3.99; 95%CI: 1.81–8.84) remained significantly more likely to have been circumcised on multivariate analysis **(**Table 4**)**.

**Table 4:** Bivariate and Multivariate analysis of factors associated with male circumcision among males attending Form 4 in Shiselweni high schools, Eswatini, 2018 (n = 407)

		BIVARIATE			MULTIVARIATE			
Explanatory variables	Explanatory variables categories	Odds Ratio	95% Confidence Interval	p-value	Adjusted Odds Ratio	95% Confidence Interval		p- value
**Age**		1.19	1.05–1.35	0.007*	1.02	0.87–1.21		0.771
**Living with**	Other^1^	1						
	One Parent	1.02	0.56–1.87	0.952	0.97	0.51–1.88		0.944
	Both Parents	0.43	0.23–0.79	0.007*	0.35	0.18–0.69		0.002*
**School Location**	Urban	1						
	Rural	0.77	0.41–1.44	0.420				
**Knowledgeable about VMMC**	Yes	1.50	0.94–2.4	0.089	1.433	0.78–2.62		0.242
**Positive Attitude toward VMMC**	Yes	1.50	1.00–2.26	0.052	0.20	0.77–3.39		0.202
**Sexually Active**	Yes	2.05	1.00–2.26	0.001*	1.53	0.90–2.61		0.117
**Parented Children**	Yes	3.00	1.28–7.04	0.011*	3.55	1.2–10.48		0.022*
**Have a family member who is circumcised**	Yes	2.06	1.01–4.22	0.048*	2.42	0.96–6.09		0.061
**Have friends who are circumcised** **circumcised**	Yes	3.96	2.09–7.49	< 0.001*	3.99	1.81–8.84		0.001*
**At what age did you become sexually active**	21 and above	1						
	15–20	4.44	0.45–43. 53	0.201				
	10–15	3.13	0.30–32.27	0.338				
**Have ever used a condom**	Never	1						
	Always	0.71	0.40 − 1.24	0.228				
**Who is the circumcised family member**	Grandfather	0.36	0.03–4.59	0.428				
	Father	0.93	0.33–2.63	0.896				
	Brother	0.83	0.34–1.99	0.672				
	Cousin	0.65	0.24–1.81	0.413				

Note * statistically significant at p-value 0.05

^1^Other includes aunt, uncle and siblings

On bivariate analysis, participants who had a circumcised family member (cOR: 0.37; 95% CI: 0.17–0.81) were less likely to intend circumcising but this association did not persist on multivariate analysis. While not significant, participants who had circumcised brothers (cOR: 1.33) and cousins (cOR: 3.68), lived in a rural location (cOR:1.08), lived with both parents (cOR:1.14), became sexually active at a younger age and always used a condom (cOR:1.94) were more likely to accept circumcision **(**Table 5**).**

**Table 5:** Bivariate and multivariate analysis of factors associated with acceptability of voluntary medical male circumcision among males attending form 4 in Shiselweni high schools, Eswatini, 2018 (n = 206)

		BIVARIATE			MULTIVARIATE		
Explanatory variables	Explanatory variables catergories	Odds Ratio	95% Confidence Interval	p- value	Adjusted Odds Ratio	95% Confidence Interval	p- value
**Have circumcised family member**	Yes	0.37	0.17–0.81	0.013*	0.49	0.22–1.10	0.082
**Age**		0.93	0.79–1.09	0.373			
**School Location**	Urban	1					
	Rural	1.08	0.52–2.24	0.841			
**Living with**	Other1						
	One Parent	0.68	0.29–1.56	0.361			
	Both Parents	1.14	0.53–2.47	0.730			
**Knowledgeable about VMMC**	Yes	1.00	0.57–1.76	0.998			
**Positive Attitude toward VMMC**	Yes	0.75	0.44–1.26	0.27			
**Sexually Active**	Yes	0.72	0.43–1.21	0.215			
**Parented Children**	Yes	0.64	0.16–2.47	0.513			
**Have circumcised friends**	Yes	0.53	0.27–1.03	0.063			
**At what age did you become sexually active**	21 and above	1					
	15–20	3.91	0.36–41. 89	0.260			
	10–15	1.30	0.11–15.45	0.838			
**Have ever used a condom**	Never	1					
	Always	1.94	0.83 − 4.52	0.126			
**Who is the circumcised family member**	Grandfather	0.79	0.04–14.03	0.869			
	Father	0.51	0.10–2.51	0.408			
	Brother	1.33	0.37–4.75	0.658			
	Cousin	3.68	0.87–15.54	0.076			

## Discussion

The findings of this study indicated the circumcision prevalence (48.98%) was higher in this study than the recent global estimation of male circumcision which is at 37–39%, where almost half of these procedures are carried out for cultural or religious reasons [24, 25]. EmaSwati do not circumcise in the cultural context [26]. The prevalence in this study is slightly higher than a self-reported prevalence of 42.8% in a study done in South Africa [27]. It is much higher than that reported amongst Polish university students in 2017 (16.7%) [24]. This study also shows a higher prevalence than the self-reported prevalence of VMMC among youth (16.7%) in the Bahamas in 2019 [28]. However, most of the uncircumcised young men in that study were considering circumcision for HIV prevention purposes [28].

Among the uncircumcised youth in this study population, 23.43% were willing to get circumcised when they had time. This is low when compared to other countries and should be a concern for government. A study done in the Bahamas reported 35% of uncircumcised youth were considering circumcision [28] while a study done in South Africa, in 2014 reported that 45.7% of the uncircumcised youth (between age 15–24) would consider undergoing VMMC [27]. In another study done in Rwanda, 50.2% of uncircumcised men considered circumcision [29].

The median knowledge score of (75.74%) showed that they were knowledgeable. The knowledge level of young males in this study is higher than that of a Ugandan study in 2018, where it was 30.6% [30]. However, the knowledge level in this study is slightly lower than in another study done in Botswana in 2012 where it was 87% [31]. Nevertheless, we may not overlook the fact that in this study no information sharing prior to the interview was done.

Cues to action define all events, people or things that are instrumental in pushing individuals to change behaviour [20]. We assumed that knowledgeable young males are more likely to get circumcised. On multivariable analysis while not statistically significant, those who are knowledgeable were 1.5 times more likely to be circumcised, than those who were not knowledgeable.

When knowledge was tested in this study, one of the variables used was to ask if male circumcision prevents the risk of STIs. The majority of the male youth (78.86%) correctly answered the question **(**Table 2**).** This is consistent with the findings of a study done in KwaZulu-Natal [18] where it was found that knowledge can also be a facilitator of VMMC as young males may perceive hygiene advantages. In the same study adolescents perceived increased sexual pleasure if they were circumcised. Similar findings were also found in a study conducted in Johannesburg, where most participants were motivated to be circumcised to prevent STIs [32]. Having non-significant results on the HIV related variables in this study may suggest that the young males are not very clear about the benefits of VMMC on HIV.

In this study we found that 17% of the participants agreed that VMMC is very painful and may lead to death. This is lower when compared to other studies [6, 13, 14, 33]. Since Eswatini is a non-circumcising country, young males might think that like in traditional male circumcision, pain is a pre-requisite for the procedure in order to “be a man:” This may indicate existence of barriers towards VMMC amongst the young males and that includes their perceptions of sexual satisfaction. In some other studies, the existence of these barriers associated VMMC uptake with promiscuity and stigma[14, 33]. Therefore, capacity building must be applied at all levels, from individual to national level. Another study have proved that capacity building is effective [34]. Importantly, Eswatini has the highest HIV prevalence in Africa, so, interventions such as VMMC to reduce the burden are important [35]. However, negative attitudes amongst the youth to VMMC, limit uptake and result in a persistently high HIV prevalence level. Thus interventions which address the negative attitudes amongst the youth have to be implemented to improve VMMC uptake.

Some of the young males (55.5%) in this study perceived increased sexual feeling after circumcision (Table 2). A qualitative study in Tanzania showed respondents who highlighted those themes of increased sexual feelings in their interviews [36]. This corroborates with the literature, where sexual performance interest in young males was a facilitator for uptake of VMMC. Unfortunately it also became a factor for risk compensation [19]. It is more likely that once people perceive themselves to be at a lower risk of contracting HIV post VMMC, they will be less careful and will not use a condom [19].

It was interesting to note that the young males in this study had misconceptions about sexual pleasure post male circumcision. This echoes the findings of a study done in Uganda in 2018, where the concern of reduced performance was strongly associated with circumcision status [30]. In another qualitative study done in Eswatini in 2015, males did not want to undergo the procedure as they believed that it reduced sexual pleasure. They further justified their decision by mentioning that they were faithful to their partners and practised other preventive measures [37]. Nevertheless it was reassuring that adolescents in the same study were mostly positive about circumcision. They believed that girls preferred circumcised men [38]. A novel approach should be used to address these misconceptions in Eswatini. Where possible, the female partners should be included [30].

Surprisingly, adolescents with a positive attitude towards VMMC in this study were less likely to have been circumcised (Table 2**).** Factors that may lead to this include, Eswatini being a non-circumcising country and Christianity being the common religion. A study done in Rwanda, in 2012, explored the knowledge and perception of Rwandan men about VMMC to determine the factors associated with VMMC uptake. In that study, the prevalence was high among those who live in Kigali where there is a spread of the Muslim religion[39]. It was also evident in Tanzania where circumcision has increased among the non-circumcising ethnic group who have social contact with circumcising groups [36]. Therefore, it is crucial that the VMMC campaigns are modified to suit the diversity of the population and to consider other social dynamics in different areas [40]. The barriers of VMMC among that particular population should be addressed.

Living with both parents was statistically associated (p = 0.002) with the presence of circumcision on multivariate analysis **(**Table 5). However, those living with both parents were less likely to be circumcised than those who lived with a single parent or just relatives. This is contrary to the review findings done in SSA where parents thought it important to be supporting their adolescents’ in VMMC decision making. Surprisingly, those who lived with both parents were more likely (OR = 1.14) to accept circumcision than those who lived with their relatives, though not significant. A study done in China showed that parents can play a major role in male circumcision uptake, more especially among younger children [41]. The above-mentioned studies’ findings could mean a challenge and a gap in adolescent-parent communication. Some parents may not support discussions of HIV risk and sexual health information due to their cultural and gender norms, or lack of appropriate knowledge regarding HIV [42]. The findings from this study show that parents’ contribution to the cue to action construct of the HBM being significantly associated with VMMC acceptance. However, since there is a minority who lived with relatives who were less likely to be circumcised, there is a need for a social marketing strategy to both the young males in schools and parents too. This is more especially because VMMC is just a new preventative measure for HIV and the parents are not familiar with its evidence.

### Sexual activity

The majority of young males in this study (57.28%) reported being sexually active and they were 1.5 times more likely (OR = 1.53) to be circumcised than those who are not. This is similar to a study done in China, 2016, where the perceived susceptibility construct was statistically significant. It is however concerning, that almost half of the sexually active young males (48.9%) did not always use a condom during sexual intercourse. The HIV prevalence in Eswatini is very high (27%) and this risky behaviour exposes them to HIV and sexually transmitted infections (STIs) [35].

The higher odds of those who are sexually active being circumcised, may increase HIV and STI infection risk if they do not use a condom. These results suggest that VMMC counselling for both sexually active and abstinent adolescents needs to be substantially improved to increase knowledge and post-VMMC preventive intentions against sexual risk. According to the literature, female partners, especially non-married, may have the ability to negotiate safer sex thus influencing their male partners to be circumcised [43]. Moreover, unlike in the past, women nowadays can play a major role in sexual matters within the relationships. Females might also have knowledge of being prone to cervical cancer when engaging with an uncircumcised partner [43, 44].

From these results, it is shown that HBM is a potentially suitable framework to guide the design for future interventions to upscale VMMC in order to reduce HIV infection. This should increase the perceived susceptibility and perceived severity related to HIV. Other approaches besides information dissemination are warranted. The fear appeal approach can be used as a useful strategy. According to this approach, a perceived threat is triggered only in the presence of both perceived susceptibility and perceived severity [45]. However, it should be applied in an encouraging way and in an ethical manner. This application is necessary since in this study, those who do not use a condom are less likely to be circumcised (OR = 0.44), suggesting that they do not fear contracting HIV.

In this study, those who parented children were three times more likely (OR = 3.55) to be circumcised than those who do not have children. As a father, you are considered a major influence in the household’s decision-making process and because of the power dynamics, if they undergo VMMC they can be role models to their sons [46]. This correlates with a study in Kenya which showed 90% of circumcised fathers being role models to their adolescent sons. They believed their experience can lead others, including their sons, to understand the impact of VMMC in preventing HIV and to seek out the procedure for their own health [47].

Behavioural change at an individual level may be influenced by interpersonal communication between family members and friends. People’s experiences are likely to change other people’s perceptions and attitudes towards health interventions like VMMC, thus increasing the uptake. This study showed that adolescents who had friends who were circumcised were almost four times more likely to be circumcised than those who had uncircumcised friends. In a study conducted in Zimbabwe in 2014, males who reported to have social support for VMMC from their friends were three times more likely to get circumcised [33].

The results from this study have shown that peer norms can influence adolescent and youth decisions. Some adolescent boys may fear stigma from peers and girls if they are not circumcised. However, some may be less concerned about stigma but just respond to external cues such as seeing their friend volunteering for the procedure.

### Limitations

Information bias could be the possible limitation of this study as we relied on self-reporting on their circumcision status. The boys may have reported in the affirmative in order to be seen as displaying the correct behaviour. The quantitative nature of the study channelled the participants to answer according to the data collection tool. They could not express themselves further. The questionnaire did not ask when and why they were circumcised.

Since it is a cross-sectional study design, we measured responses at one point in time which might affect study results as the participants could have responded depending on their mood, feelings or other stimulants at that particular time. It might happen that if the participants were given the same questionnaire at a different time and setting, that they would not give the same responses. Further, answering the questionnaire while the researcher waited could have created pressure on the participants and hence affected their responses.

## Conclusion

The results of this study have shown that although the young males have knowledge about VMMC, they have a negative attitude towards it. Neither knowledge nor attitude were associated with the acceptability of VMMC. The social environment influenced the uptake of VMMC amongst adolescent males in Eswatini. The findings of this study will help in improving the VMMC programme implementation in future.

## Data Availability

The datasets used in this study is available from the corresponding author on reasonable request.

## Abbreviations

AIDS: Acquired Immunodeficiency Syndrome
EMIC: Early Infant Male Circumcision
HBM: Health Belief Model
HIV: Human Immune Virus
UNAIDS: Joint United Nations Programme on HIV/AIDS
VMMC: Voluntary Medical Male Circumcision
WHO: World Health Organisation
